# Potential of Wheat Straw, Spruce Sawdust, and Lignin as High Organic Carbon Soil Amendments to Improve Agricultural Nitrogen Retention Capacity: An Incubation Study

**DOI:** 10.3389/fpls.2018.00900

**Published:** 2018-06-28

**Authors:** Rüdiger Reichel, Jing Wei, Muhammad S. Islam, Christoph Schmid, Holger Wissel, Peter Schröder, Michael Schloter, Nicolas Brüggemann

**Affiliations:** ^1^Forschungszentrum Jülich GmbH, Institute of Bio- and Geosciences, Agrosphere (IBG-3), Jülich, Germany; ^2^General and Theoretical Ecology, Institute of Ecology, University of Bremen, Bremen, Germany; ^3^Research Unit Comparative Microbiome Analysis (COMI), Deutsches Forschungszentrum für Gesundheit und Umwelt GmbH, Helmholtz Zentrum München, Munich, Germany

**Keywords:** greenhouse gases, high organic carbon amendment, isotope labeling, microbial decomposition, nitrogen immobilization, nitrogen cycle, nitrogen fertilizer

## Abstract

Plants like winter wheat are known for their insufficient N uptake between sowing and the following growing season. Especially after N-rich crops like oilseed rape or field bean, nitrogen retention of the available soil N can be poor, and the risk of contamination of the hydrosphere with nitrate (NO_3_^-^) and the atmosphere with nitrous oxide (N_2_O) is high. Therefore, novel strategies are needed to preserve these unused N resources for subsequent agricultural production. High organic carbon soil amendments (HCA) like wheat straw promote microbial N immobilization by stimulating microbes to take up N from soil. In order to test the suitability of different HCA for immobilization of excess N, we conducted a laboratory incubation experiment with soil columns, each containing 8 kg of sandy loam of an agricultural Ap horizon. We created a scenario with high soil mineral N content by adding 150 kg NH_4_^+^-N ha^-1^ to soil that received either wheat straw, spruce sawdust or lignin at a rate of 4.5 t C ha^-1^, or no HCA as control. Wheat straw turned out to be suitable for fast immobilization of excess N in the form of microbial biomass N (up to 42 kg N ha^-1^), followed by sawdust. However, under the experimental conditions this effect weakened over a few weeks, finally ranging between 8 and 15 kg N ha^-1^ immobilized in microbial biomass in the spruce sawdust and wheat straw treatment, respectively. Pure lignin did not stimulate microbial N immobilization. We also revealed that N immobilization by the remaining straw and sawdust HCA material in the soil had a greater importance for storage of excess N (on average 24 kg N ha^-1^) than microbial N immobilization over the 4 months. N fertilization and HCA influenced the abundance of ammonia oxidizing bacteria and archaea as the key players for nitrification, as well as the abundance of denitrifiers. Soil with spruce sawdust emitted more N_2_O compared to soil with wheat straw, which in relation released more CO_2_, resulting in a comparable overall global warming potential. However, this was counterbalanced by advantages like N immobilization and mitigation of potential NO_3_^-^ losses.

## Introduction

Global demands for nitrogen (N) in crop production have been 110 million tons in 2015. N demands are expected to increase to almost 119 million tons in 2020 (Lu and Tian, 2017). About one third of this amount is not directly assimilated by plants and, if not stored in the pedosphere, contaminates the hydrosphere with nitrate (NO_3_^-^) and the atmosphere with nitrous oxide (N_2_O) and nitric oxide (NO) (Zhu and Chen, 2002). N retention is of particular importance when residues of N-rich crops, such as field bean (*Vicia faba* L.), oilseed rape (*Brassica napus* L.), sugar beet (*Beta vulgaris* L.), and potato (*Solanum tuberosum* L.) are mineralized in soil. Such residues are substantial sources of mineral N, supplying between 20 and 60 kg N ha^-1^ to the soil, depending on the leaf and straw yield (Döhler, 2009). Crop residues with a C:N ratio below 20–25 are supposed to be mineralized more quickly than those with higher C:N ratios (Mooshammer et al., 2014). Sugar beet residues, for instance, with a narrow C:N ratio of 11 can be mineralized by up to 75% within the first 10 weeks after incorporation (Whitmore and Groot, 1997).

In German crop rotations, oilseed rape is harvested already in July, often followed by winter wheat (*Triticum aestivum* L.), which is sown in September/October, but grows only very slowly until the following spring, associated with a correspondingly low N uptake of up to 30 kg N ha^-1^ during this period of time (Sieling et al., 1999). In contrast, soil mineral N content after oilseed rape harvest sometimes exceeds 100 kg N ha^-1^ (Henke et al., 2008). As a result, about 50% of mineral N derived from decomposition of N-rich crops like oilseed rape can be lost from soil, particularly in sandy soil (Döhler, 2009). Sieling and Kage (2006) reported annual soil mineral N losses via nitrate leaching of 44 or 73 kg N ha^-1^ after oilseed rape harvest and subsequent winter barley (*Hordeum vulgare* L.) or winter wheat cultivation from Germany. Annual N losses by N_2_O from long-term field experiments with oilseed rape in Germany ranged between 1.4 and 4.0 kg N ha^-1^, of which 53 to 81% occurred during the winter season (Kaiser and Ruser, 2000). Thus, managing soil mineral N after harvest during times without sufficient winter crop N uptake is of great importance for reducing N losses and improving the agricultural N use efficiency (NUE) by achieving a similar quantity of N in the harvested crop by less N input (Zhang et al., 2015). Although this fact has been known for a long time, the nitrogen surplus is a persistent and pressing problem. Thus research efforts are needed to manage the fate of unused fertilizer N in crop production (Liu et al., 2010). Closing the post-harvest time gaps in crop rotations without N uptake by plants may help to retain N in soil for subsequent crop production.

It is known since long that N-poor crop residues such as wheat straw can cause a strong immobilization (or “lock”) of N, which means a reduction of plant-available N. It has been shown that available N in soil is immobilized after application of decomposable, C-rich organic residues with large C:N ratios, such as wheat straw (Richards and Norman, 1931; Cheshire et al., 1999), which might lower crop yield in the next season. Malhi et al. (2011), however, proposed that well managed soils will benefit in the long term from retaining straw residues in the soil, thereby improving crop yield and plant N uptake. Nonetheless, if N immobilization interferes with plant growth, the probability is high that microorganisms are more competitive for nutrients than plants if plant and microbial N uptake occur simultaneously in the same soil volume (Hodge et al., 2000).

Organic substrates with large C:N ratios, such as wheat straw (C:N 50–100), are required to promote microbial N immobilization, forcing microbes to take up N from soil, in order to maintain their C:N ratio at least ten times lower (Cleveland and Liptzin, 2007; Scheller and Joergensen, 2008). However, large C:N ratios alone do not warrant intensive microbial growth and thus N immobilization, but serve as a rough indicator for its N immobilization potential. Spruce sawdust with a C:N ratio of 100 to 400 contains more recalcitrant compounds, leading to lower degradation rates and prolonged times of decay (Kostov et al., 1991). Thus, in order to immobilize N, two major requirements have to be fulfilled to stimulate growth of microbial decomposers: (1) sufficient available C as energy source; (2) sufficient available N and P to fulfill stoichiometric requirements and to avoid nutrient mining of original soil organic matter (Fontaine et al., 2004).

High organic carbon amendments (HCA) consist of very different quantities and qualities of fast, moderately, and slowly degradable organic sub-fractions. Microorganisms respond very differently to these organic fractions, and microbial N immobilization can be expected to increase with increasing size and C:N ratio of the fast degradable fraction, which usually is degraded within a few days (Plante and Parton, 2007). The fast degradable fraction contains easily soluble sugars, amino acids, and nucleic acids (Müller et al., 1998). Such compounds are favorable for a rapid growth of zymogenous (fermentative) microorganisms (Kuzyakov and Blagodatskaya, 2015) with short half-lives of a few days (Cochran et al., 1988). Despite this fast turnover, C and N released by dead microbial biomass serve as additional substrates for microbes that grow more slowly on more complex organic compounds (Fontaine et al., 2004).

More complex, polymeric components such as cellulose and hemicellulose are the dominating fraction of wheat straw. Investment in depolymerizing enzymes may reduce the microbial growth efficiency, but the large C:N ratio of this fraction and its accessibility to many soil microbes account for its N retention capacity (Plante and Parton, 2007). Woody sawdust contains more recalcitrant compounds than wheat straw, mainly in the form of lignin. Specialists like white-rot fungi are needed to break up the lignified structures of wood (van Kuijk et al., 2017). However, this process is too slow to support significant microbial growth and, hence, fast microbial N immobilization. In addition to biotic N immobilization and N adsorption in soil, also less prominent ways of abiotic N immobilization were reported (Miyajima, 2015), e.g., the reaction of nitrite with HCA-derived lignin or its derivatives (Wei et al., 2017).

The aim of the present work was to test the potential of different HCA for efficiently managing a post-harvest excess of soil mineral N in arable soil without sufficient plant N uptake. To this end, a static laboratory incubation study was conducted to answer the following research questions: (1) Can HCA with large C:N ratio such as wheat straw, spruce sawdust, and lignin help to manage temporal N excess in agricultural soil by inducing microbial growth at a relevant magnitude? (2) Can such HCA application strategies help improve the nitrogen retention capacity of the soil by reducing an unintended N loss in form of nitrate and greenhouse gases?

## Materials and Methods

### Soil and HCA

For the incubation study, substrate of a Cambic Luvisol soil type with sandy loam soil texture, containing 21% clay and 35% silt in the dry matter (dm) was used, sampled in summer of 2016 from the Ap horizon at the Hohenschulen experimental site, Achterwehr field (54°19′05″N, 9°58′38″E), Kiel, Germany. In 2015, 1 year before sampling, last crops were a mixture of catch crops like red clover (*Trifolium pretense* L.) and alfalfa (*Medicago sativa* L.) without any fertilization. In 2014, 2 years before sampling, maize (*Zea mays* L.) was grown, with typical slurry and triple superphosphate fertilization.

Results were recalculated on a kilogram per hectare basis using the following input parameters of the field site: 10,000 m^2^ × 0.2 m soil depth × bulk density of 1,500 kg m^3^ = 3⋅10^6^ kg dry soil ha^-1^. The air-dried, sieved (Ø 2 mm), and homogenized soil had the following basic characteristics: pH (CaCl_2_) = 6.0 ± 0.1, organic carbon (*C*_org_) = 1.3 ± 0.1%, total *N* = 0.15 ± 0.01%.

Wheat straw was also obtained from the Hohenschulen experimental site (Kiel, Germany) with the following characteristics: *C* = 44.4 ± 0.1%, *N* = 0.28 ± 0.02%. Spruce sawdust was obtained in 2015 from Holz Ruser (Bornhöved, Germany): *C* = 45.8 ± 0.2%, total *N* = 0.06 ± 0.01%. Alkali lignin was obtained from VWR (Germany) *C* = 61.6 ± 0.1%, *N* = 0.43 ± 0.01%, suspended in water and sieved to obtain particulate pure lignin. Organic substrates were applied at a rate of 1.5 g C kg^-1^ soil, equivalent to a field application of 4.5 t C ha^-1^. In total, wheat straw, spruce sawdust, and lignin treatments received 3.4, 3.3, and 2.4 g dm kg^-1^. At the beginning of the pre-incubation period, 90% of the HCA were mixed homogeneously with the soil and 10% was buried horizontally at a soil depth of 2 cm in litter bags made of 0.2 μm nylon meshes, a soil contact area of 36 cm^2^, and an average thickness of 0.25 cm after filling (Supplementary Figure S1). The particle size of the HCA applied to soil and litter bags ranged around 1 mm.

### Experimental Setup

A combined application of N fertilizer and organic application was used for the wheat straw (SWF = Soil + Wheat straw + Fertilizer), spruce sawdust (SSF = Soil + Spruce sawdust + Fertilizer), and lignin (SLF = Soil + Lignin + Fertilizer) treatments. The control treatment (S = Soil) did not receive any fertilizer or HCA, the fertilizer control treatment (SF = Soil + Fertilizer) received only N fertilizer, but no HCA. Each treatment consisted of three independent replicates. Custom-made stainless steel incubation columns with a height of 30 cm, a diameter of 20 cm, and a detachable, unsegmented headspace unit were constructed by the workshop of Forschungszentrum Jülich, Germany (Supplementary Figure S1). On average, 8,000 cm^3^ of headspace volume were achieved after connecting the headspace unit to the column, considering individual differences of each soil column, e.g., slightly deviating soil levels. Airtightness was achieved by a silicone rubber O-seal, embedded in cut grooves of the headspace and incubation column before screwed in place. Gas-tightness of the system was checked before the experiment, using helium and a helium detector.

**FIGURE 1:**
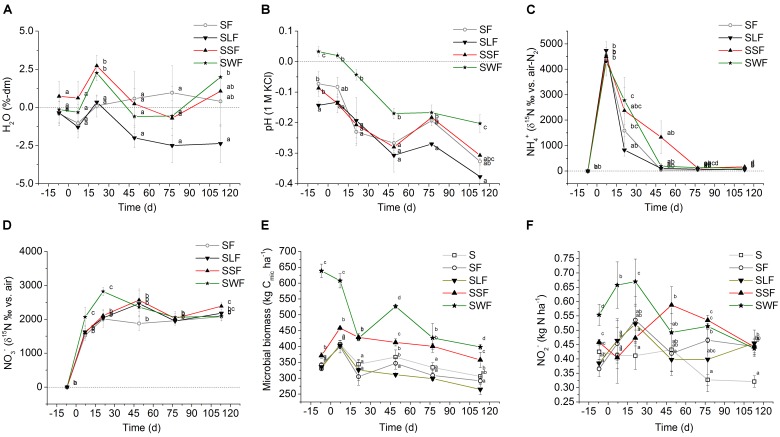
**(A)** H_2_O dynamics (%-dm, dry soil), **(B)** pH development (1 M KCl soil extract), **(C)** ^15^NH_4_^+^ (δ^15^N ‰ vs. air-N_2_), and **(D)** ^15^NO_3_^-^ (δ^15^N ‰ vs. air-N_2_) in soil of the fertilizer control treatment (SF, open circle) with mineral N fertilizer, and the HCA treatments with mineral N fertilizer plus lignin (SLF, solid triangle downward), spruce sawdust (SSF, solid triangle upward), or wheat straw (SWF, solid star symbol) relative to the control treatment (S, *y* = 0) without any fertilizer or HCA. **(E)** microbial biomass C (kg *C*_mic_ ha^-1^) and **(F)** nitrite concentration (NO_2_^-^ μg N ha^-1^) in soil of the control treatment (S, open square) without any fertilizer or HCA, the fertilizer control treatment (SF, open circle) with mineral N fertilizer, and the HCA treatments with mineral N fertilizer plus lignin (SLF, solid triangle downward), spruce sawdust (SSF, solid triangle upward), or wheat straw (SWF, solid star symbol). Incubation time *d* = 0 divides the experiment into a period before (–7 DBF) and after (7, 21, 49, 77, and 113 DAF) mineral N fertilization. Standard deviations of mean values (*n* = 3) are displayed. Statistically significant differences between treatments at a certain incubation time are depicted by different lowercase letters next to the symbols (*p* < 0.05).

Each soil column contained six equally spaced sections made of polyvinyl chloride (PVC) (Supplementary Figure S1), allowing sampling in time intervals without substantial disturbance of the soil structure. Void spaces after sampling where filled with solid PVC spacers to keep the gas sampling headspace at constant volume. 8 kg of soil was mixed with or without HCA before filling equal amounts of soil in each column section. Bulk density of the soil was adjusted to the field value of 1.5 g cm^-3^ by using a special triangle-shaped tool that exactly fit into the column section. During the first 12 h of the pre-incubation period of 7 days before fertilizer application (DBF), soil was rewetted to a water volume equivalent of 40% of the water holding capacity measured in soil without HCA (WHC, 35 g H_2_O 100 g^-1^ dm). Deionized H_2_O was dripped onto the soil surface in steps of maximal 50 ml to re-activate soil microorganisms before adding fertilizer N. To simulate an excess of soil mineral N, ammonium sulfate (NH_4_)_2_SO_4_ was applied as fertilizer with a ^15^N content of 2.65 atom-% (corresponding to a δ^15^N of about 6,400‰; VWR, Germany). The N fertilizer, equivalent to 50 mg N kg^-1^ soil and to 150 kg N ha^-1^, was dissolved in 100 ml deionized H_2_O to prepare a stock solution. After further dilution with deionized H_2_O, the N solution was poured stepwise onto the soil surface of the fertilized treatment, finally adjusting the water content to 60% WHC. Soil substrates of the control treatment (S) only received deionized H_2_O instead of N solution. Soil columns were incubated over a total of 120 days at room temperature from 11th November 2016 to 16th March 2017. Average temperatures during incubation ranged from 20.3 to 22.3°C. Soil moisture loss was monitored between the sampling dates by weighing the soil column, and gravimetrically after each sampling event. Soil moisture was kept in a range of 50 to 60% WHC by dripping deionized H_2_O equivalent to 7.5 ml d^-1^ onto the soil surface of each sampling segment. As indicated in **Figure 1A**, average water contents were at a comparable level of ± 1%-dm in all treatments after application of H_2_O with and without N at 7 DBF and 7 days after fertilizer application (DAF), despite anticipated effects of organic amendments on the WHC.

### Soil Sampling and Analyses

Spatulas and spoons with prolonged shafts were used to sample soil equivalent to 1.3 kg dm from each column section without disturbance of the remaining ones. Seven days prior to N fertilizer application, the first soil column section was sampled as reference. All other soil samplings were conducted at 7, 21, 49, 77, and 113 DAF. Soils for chemical analyses were immediately frozen, freeze-dried and stored in plastic bags at room temperature. Litter bags with HCA were treated the same way, but additionally were cleaned by gentle brushing, before HCA decomposition was calculated by determining the weight loss between applied and recovered HCA dry matter. Fresh soil was directly stored after sampling at -20°C for later determination of microbial biomass, and at -80°C for PCR analysis of the major N cycling genes.

Microbial biomass carbon (*C*_mic_) was determined after chloroform fumigation and extraction with 0.01 M CaCl_2_ solution as described in Reichel et al. (2017). *C*_mic_ was calculated using a fraction of 0.45 as extractable part of microbial biomass C (kEC). We did not apply pre-extraction of fresh soil with 0.5 M K_2_SO_4_ to remove the large inorganic N background as recommended by Widmer et al. (1989) or Wachendorf and Joergensen (2011), which may have confounded measurements of N retained in the microbial biomass (*N*_mic_). We then decided to calculate the *N*_mic_, using a calculated average *C*_mic_:*N*_mic_ ratio of 7, which was reported for comparable agricultural soils with organic C amendments by Scheller and Joergensen (2008).

Abundance of the ammonia oxidizing bacteria (AOB) and archaea (AOA), as well as of various denitrifiers, was calculated based on marker genes quantified by real-time quantitative polymerase chain reaction (qPCR) on selected samples taken at 7, 49, and 113 DAF. For ammonia oxidizers the *amoA* gene coding for an ammonia monooxygenase was used as marker; for denitrifiers nitrate reductase (*narG*), nitrite reductases (*nirK* and *nirS*), and nitrous oxide reductase (*nosZ*) genes were determined. In short, DNA was extracted from 0.5 g of fresh soil using the NucleoSpin Soil Kit (Macherey-Nagel GmbH & Co. KG, Düren, Germany) according to the manufacturer’s protocol. Dilutions (1:16 v/v) of the raw extracts were used for quantification by (qPCR) with the 2X Takyon for SYBR Assay master mix (Kaneka Eurogentec S.A., Seraing, Belgium). To reduce the inhibitory effect of polyphenolic compounds co-extracted from soil, we added bovine serum albumin (BSA) to a final concentration of 0.06% to each reaction and 2.5% dimethyl sulfoxide (DMSO) to reactions involving the *nir* genes. All reactions were run in triplicate on a 7300 Real-Time PCR System (Applied Biosystems, Foster City, CA, United States). The PCR program involved an initial activation step at 95°C for 3 min, followed by 39 cycles at 95°C for 10 s, primer melting temperature (T_M_) for 20 s, 72°C for 45 s. T_M_ corresponded to 55°C for *AOA*, 60°C for *AOB*, 63°C for *narG* and *nirK*, 57°C for *nirS* and 65°C for *nosZ*. Reaction specificity was checked using a melting curve analysis. The copy number was calculated from a standard curve of serial 10-fold dilutions of plasmids containing the target gene in known concentrations (Ollivier et al., 2010). More details about the gene primer sequences of AOB-*amoA, AOA-amoA, nirS, nirK*, and *nosZ* are available in Zhang et al. (2013), and of *narG* in Bru et al. (2007). Gene copies per gram soil were normalized to *C*_mic_ (gene copies per μg *C*_mic_).

Soil pH was determined with a pH meter (multi 340i, WTW GmbH, Weilheim, Germany) according to the ISO 10390 method (ISO, 2005) using 1 M KCl solution at a soil:solution ratio of 1:5 (w/v) and mixed for 2 h.

High organic carbon soil amendments residues of litter bags were extracted three times with 1 M KCl solution at a ratio of 1:5 (w/v), before freeze-dried HCA residues were analyzed for N content and ^15^N isotope signature, using an isotope-ratio mass spectrometer (IRMS, Delta V plus, Thermo Fisher Scientific, Bremen, Germany). Soil NH_4_^+^ and NO_3_^-^ content was determined by applying sequential micro-diffusion and liquid-liquid extraction techniques (Mulvaney et al., 1997; Huber et al., 2012): 80 ml 1 M KCl solution (the solid KCl had been pre-treated by heating for 16 h at 550°C to minimize background NH_4_^+^ prior to preparation of the KCl solution) was mixed with 8–9 g soil, shaken at 200 rpm for 1 h, centrifuged for 20 min at 3000 rpm, and filtrated through Whatman no. 42 filter paper with 2–3 μm pore size. 60 ml of the KCl extract was transferred to 100-ml polypropylene (PP) bottles, and the pH was adjusted to about 12, using 1 M NaOH solution, to convert NH_4_^+^ to NH_3_. NH_3_ was allowed to volatilize at room temperature for 7 days and was collected with 2 × 15 μl of saturated oxalic acid pipetted onto quartz glass filter disks. Afterward, the disks were transferred to a desiccator and dried over silica gel for 24 h. Then, the dry filter disks were packed in tin (Sn) capsules and analyzed using an elemental analyzer coupled to an IRMS (EA-IRMS, Flash EA 2000 and Delta V Plus; Thermo Fisher Scientific, Bremen, Germany). The remaining KCl extract was dried at 65°C, re-dissolved in 3 ml of 1 M NaOH, and mixed with 37 ml acetone for 30 s. After centrifugation at 3,000 rpm for 20 min, the acetone supernatant containing most of the NO_3_^-^ was transferred into glass beakers, dried at 30–40°C, re-dissolved in 5 ml of deionized water, freeze-dried, and finally transferred into Sn capsules for EA-IRMS analysis (Zhu et al., 2013). Nitrite was monitored as secondary parameter, using an improved colorimetric method according to Colman (2010) and Homyak et al. (2015). A DU-800 spectrophotometer (Beckman Coulter, Fullerton, United States) at a wavelength of 560 nm was used for measurement.

### Gas Sampling and Analysis

CO_2_ and N_2_O emission was measured on average two to three times a week, using PVC chambers 20 cm in height and 20 cm in diameter, with a gas sample port with septum and a 100 cm long vent tube with 0.5 cm in diameter, equipped with an air-tight clamp. PVC headspace units were installed gas-tight on top of the column before gas sampling (Supplementary Figure S1). After mounting the headspace unit, a gas volume of 35 ml was sampled with a syringe through the septum of the sampling port at 0, 20, 40, and 60 min between 7 DBF and 49 DAF, increasing the interval to a maximum of 240 min at 113 DAF. The clamp on the inlet tube was opened to allow pressure equilibration during each gas sampling. These intrusions of ambient air were considered before calculating the gas emission (Equation 1). The gas samples were transferred completely to 22.5-ml pre-evacuated gas chromatography vials, thereby creating an overpressure for a proper processing by the GC autosampler during analysis. N_2_O and CO_2_ analyses were performed with a gas chromatograph, equipped with an electron capture detector and a flame ionization detector (GC-ECD/FID, Clarus 580, PerkinElmer, Rodgau, Germany). CO_2_ and N_2_O emission was calculated from the increase of corrected gas concentrations as follows:

$$F=\frac{slope(C_{1}:C_{4},t_{1}:t_{4})\times V\times K\times M}{m\times (K+T)\times Vm}$$

*F*: gas emission flux; *slope*: change of gas concentration per unit time, gas concentrations one to four (*C*_1_:*C*_4_) in ppmv for CO_2_ and ppbv for N_2_O at sampling time interval one to four (*t*_1_:*t*_4_) in hours; *V*: headspace volume in liter; *m*: amount of soil in gram dry weight; *Vm*: molar volume of ideal gases (22.414 l at 0°C and 101.325 kPa), corrected for the gas sample temperature using *K* (273.15 Kelvin) and *T* (air temperature in °C); *M*: molar mass of N in N_2_O or C in CO_2_, respectively.

Total global warming potential (GWP) was calculated as the sum of direct CO_2_-C emissions and CO_2_-C equivalents of N_2_O emissions, using a factor of 265 for the conversion of N_2_O in CO_2_ equivalents (IPCC, 2014), divided by 28 (atomic mass of the two nitrogen atoms of N_2_O) and multiplied by 12 (atomic mass of one carbon atom) to convert 1 kg N_2_O-N into 1 kg CO_2_-C equivalents.

### Statistical Analysis

We used three independent replicates, i.e., separate incubation columns, for each of the treatments (S, SF, SSF, SWF, and SLF) (Supplementary Figure S1). Furthermore, each soil column contained six equally spaced, independent sections, which allowed separate soil samplings from each column at incubation time 7 DBF and 7, 21, 49, 77, and 113 DAF (Supplementary Figure S1). Since *n* = 3 is low to reliably test normal distribution and inhomogeneity of variance, we decided to test for significant differences between treatments at certain sampling times by using an analysis of variance (ANOVA) with the Tukey-B or Games-Howell *post hoc* test, which is less susceptible to inhomogeneous variances and non-normality (Janssen and Laatz, 2010). The significance threshold value for the comparisons was set at *p* = 0.05. Statistical software used was Origin 2015 (Originlab Corporation, Wellesley Hills, MA, United States) and SPSS Statistics 20.0 (IBM Deutschland GmbH, Ehningen, Germany).

## Results

### Soil Physical Parameters

#### Water Content

Watering caused fluctuations of the gravimetric soil moisture within a range of ± 2.5% (**Figure 1A**). The lignin treatment (SLF) tended to lower H_2_O content in relation to all other treatments. There was only one significant difference between the SLF and SWF treatment at 113 DAF.

#### pH

Relative to the control treatment, the pH of other treatments slightly, but significantly decreased after fertilization with NH_4_^+^ (**Figure 1B**). In soil with wheat straw (SWF), the decrease in pH after mineral N fertilization was less pronounced compared to all other treatments.

### High Carbon Soil Amendment Decomposition and Fate of N

#### HCA Decomposition

**FIGURE 2:**
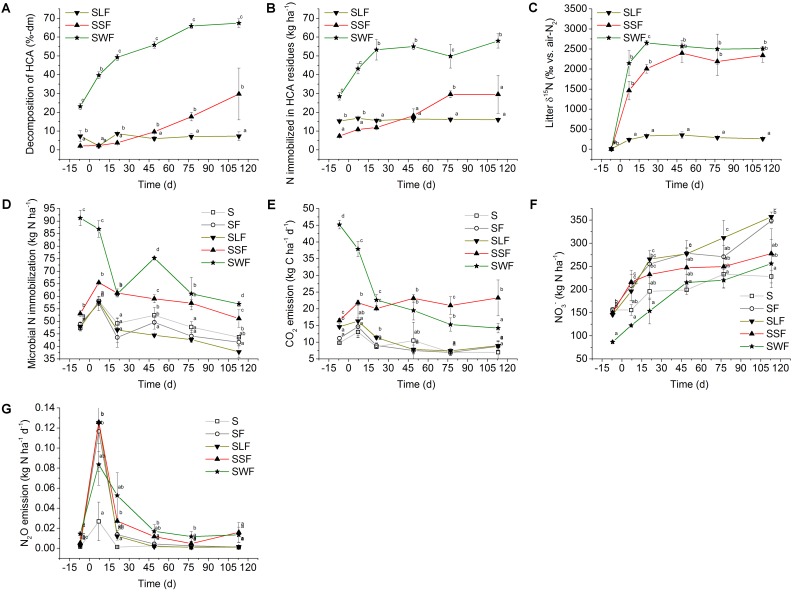
**(A)** HCA decomposition (% loss of initially applied HCA dry matter), **(B)** physical and/or chemical N immobilization by the remaining HCA residues in kg N ha^-1^ and **(C)** δ^15^N values in ‰ vs. air-N_2_ in soil treated with mineral N and lignin (SLF, solid triangle downward), spruce sawdust (SSF, solid triangle upward), and wheat straw (SWF, solid star symbol). **(D)** Microbial N immobilization (kg N ha^-1^), **(E)** CO_2_ emission 24 h before soil sampling in kg C ha^-1^ d^-1^, **(F)** NO_3_^-^ concentration in kg N ha^-1^, and **(G)** N_2_O emission in kg N ha^-1^ d^-1^ in soil of the control treatment (S, open square) without any fertilizer or HCA, the fertilizer control treatment (SF, open circle) with mineral N fertilizer, and the HCA treatments with mineral N fertilizer plus lignin (SLF, solid triangle downward), spruce sawdust (SSF, solid triangle upward), or wheat straw (SWF, solid star symbol). Incubation time *d* = 0 divides the experiment into a period before (–7 DBF) and after (7, 21, 49, 77, and 113 DAF) mineral N fertilization. Standard deviations of mean values (*n* = 3) are displayed. Statistically significant differences between treatments at a certain incubation time are depicted by different lowercase letters next to the symbols (*p* < 0.05).

Overall, only HCA of the SSF and SWF treatments showed a clear decomposition trend (**Figure 2A**). The rate of wheat straw decomposition was characterized by three phases: 0.9% dry mass loss d^-1^ (0–21 DAF), 0.3% dm loss d^-1^ (21–77 DAF), and 0.04% dm loss d^-1^ for the remaining incubation period. At the end of the experiment, 70% of the initial wheat straw dry mass was decomposed. In contrast, no clear decomposition trend occurred in the SSF treatment until 49 DAF. While the lignin treatment showed no change in degradation, the spruce sawdust decomposition after 49 DAF proceeded linearly at a rate of 0.3% d^-1^. Spruce sawdust showed a total decomposition around 30% dm in the end.

#### N Immobilization by HCA Residues

Only N that was not extractable by a combination of 1 M KCl solution and two H_2_O washing steps was considered. The application of wheat straw added 28 kg N ha^-1^ to the soil. The remaining wheat straw residues became further enriched in N during the incubation period after mineral N fertilization, particularly during the period with the fastest decomposition until 21 DAF. N in wheat straw residues increased to 53 kg N ha^-1^, retaining an extra of 25 kg N ha^-1^ (**Figure 2B**).

In contrast, only 7 kg N ha^-1^ were added with the spruce sawdust to the soil, thus about four times less compared to wheat straw. The N content associated with spruce sawdust residues increased the most between 21 and 77 DAF to 30 kg N ha^-1^, reaching a plateau afterward. Despite a slower and 40% lower degree of decomposition compared to wheat straw, N enrichment of the spruce sawdust residues reached comparable levels, retaining an extra amount of 23 kg N ha^-1^ at 77 DAF and likely also at 113 DAF, but variability impeded a significant differentiation from the SLF treatment at this incubation time. Hence, spruce sawdust and wheat straw both helped to immobilize similar N amounts (on average 24 kg N ha^-1^). Due to the higher N content of the technically processed lignin, application initially added 15 kg N ha^-1^ to the soil, containing more N in HCA compared to the SSF treatment at 7 DBF and 7 DAF. N enrichment of lignin was low and not significant over the experimental period, ranging at 1 kg N ha^-1^.

#### δ^15^N of HCA

δ^15^N values were determined in the same washed HCA-fractions (**Figure 2C**). The original δ^15^N values of HCA were 8.0‰ vs. air-N_2_ for the synthetic lignin, 3.2‰ for spruce sawdust, and 3.1‰ for wheat straw. The δ^15^N values of the wheat straw and the spruce sawdust increased to over 2,000‰ until 21 DAF. The δ^15^N of lignin, which showed only weak decomposition and N dynamics, was only enriched to maximum values of about 300‰ over the course of the incubation experiment.

### HCA-Induced Changes in the Soil Microbiome

#### N Immobilization by the Microbial Biomass

Microbial N retention per hectare and microbial biomass values (kg *C*_mic_ ha^-1^) are shown in **Figures 1E**, **2D**, respectively. Amending soil with wheat straw (SWF) instantaneously increased the microbial biomass by 309 kg *C*_mic_ ha^-1^ (corresponding to 42 kg *N*_mic_ ha^-1^) relative to the S treatment without mineral N or HCA at 7 DBF. Generally, adding H_2_O without or with mineral N significantly increased most *C*_mic_ values, except in soil with wheat straw. *C*_mic_ values of all treatments decreased over the course of the incubation experiment (**Figure 1E**). In the end, *C*_mic_ values of the SSF and SWF treatment still remained larger than in all other treatments. Starting from 7 DAF, the rapid increase in *C*_mic_ after wheat straw application was followed by a decrease. Between 7 DBF and 21 DAF, microbially immobilized N in the SWF treatment was released at a rate of 1.6 kg N ha^-1^ d^-1^. Afterward, microbial N immobilization showed a second maximum at 49 DAF with an immobilization of 26 kg N ha^-1^ relative to the SF treatment (**Figure 2D**). This again was followed by a release of N from the microbial biomass at a rate of 0.5 kg N ha^-1^ d^-1^. Relative to the SF treatment, microbially immobilized N in the SWF treatment finally amounted to 15 kg N ha^-1^ (113 DAF). About 8 kg N ha^-1^ were immobilized by the microbial biomass of the SSF treatment relative to the SF treatment at 7 DAF. Afterward, microbial N release also occurred in the SSF treatment, but at a low rate of 0.3 kg N ha^-1^ d^-1^ compared to the SWF treatment (**Figure 2D**). After 4 months of incubation, spruce sawdust still had immobilized about 9 kg N ha^-1^ in the HCA-derived microbial biomass relative to the SF treatment, which was significantly lower than in the SWF treatment, but clearly larger compared to the lignin (SLF) treatment (**Figure 2D**).

#### Influence of HCA on the Abundance of Nitrifiers and Denitrifiers

**FIGURE 3:**
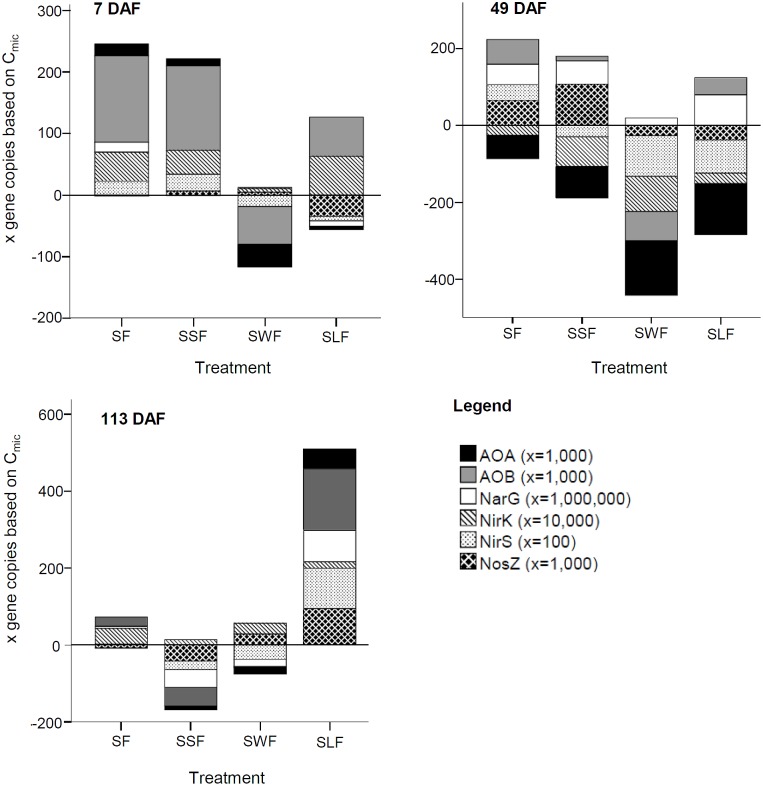
Influence of HCA on the abundance of the ammonia oxidizing bacteria (AOB) and archaea (AOA), as well as of various denitrifiers (NarG, nirK, nirS, and NosZ) at 7, 49, and 113 days after mineral N fertilization (DAF) in soil with only mineral N fertilizer (SF), with N fertilizer plus lignin (SLF), spruce sawdust (SSF), or wheat straw (SWF) relative to the control treatment S (*y* = 0) without any N fertilization or HCA application. Gene copies per gram soil were normalized to *C*_mic_ (gene copies per μg *C*_mic_). Multiplication factors of gene copies per μg *C*_mic_ are available in the according brackets next to the gene symbol and name. Statistical significances are described in the Section “HCA-Induced Changes in the Soil Microbiome” of the manuscript.

N application to soil without (SF) or with litter (SSF, SWF, and SLF) induced specific changes in the abundances of AOA and AOB as well as of denitrifiers (*narG*, *nirK/S*, and *nosZ*) relative to *C*_mic_ (**Figure 3**). N application to soil without HCA (SF) and with spruce sawdust (SSF) significantly increased the abundance of *AOA* and *AOB* at 7 DAF. Wheat straw of the SWF treatment significantly lowered the abundance of *AOA* and *AOB* at 7 and 49 DAF, relative to the unfertilized control (S). To some extent, the SF, SSF, and SLF treatment also lowered the *AOA* abundances, but only at incubation time 49 DAF. Also the abundance of denitrifers harboring the nirS gene was significantly lower in the SWF compared to the SF treatment. Wheat straw and lignin significantly lowered the abundance of denitrifiers harboring the *nosZ* genes compared to the SF and especially to the SSF treatment at 49 DAF. Trends on the abundance of nitrifiers and denitrifiers were identified also in soil of the SF and SLF treatment, but showed no statistical significance compared to the S treatment. All analyzed functional groups returned to the level of the S treatment until 113 DAF, with the exception of the SLF treatment (**Figure 3**, 113 DAF). Water content and pH are strong drivers of *C*_mic_ development, which likely decreased the functional gene abundances in the SLF treatment less than the abundance (*C*_mic_) of other microorganisms without those genes (**Figures 1A,B,E**).

### Effect of HCA on CO_2_ and N_2_O Emissions and on Soil NO_3_^-^

#### CO_2_ Emissions

CO_2_ emissions were calculated for a time interval of 24 h before soil sampling (**Figure 2E** and Supplementary Figure S2). Additionally, the cumulative CO_2_ emissions were calculated by integration of the area below the curve within the time interval between 7 DBF and 113 DAF (Supplementary Figure S2). During the phase with the largest wheat straw decomposition rates (**Figure 2A**), significantly larger CO_2_ emissions were found in the SWF treatment compared to all other treatments. After N application, CO_2_ emissions decreased until 113 DAF, which was in accordance with the microbial biomass development of the SWF treatment (**Figure 1E**). CO_2_ emissions from the SSF soil showed a delayed increase at 7 DAF and then remained at a comparable level over the entire incubation period. This is in contrast to the SWF treatment, which later significantly decreased to a lower CO_2_ emission rate comparable to the S treatment at 113 DAF. CO_2_ emitted from N-fertilized soil without HCA and with lignin did not differ from the unfertilized control treatment (S), reflecting a low microbial activity in these treatments. Consequently, cumulative CO_2_ emissions were significantly larger in the SSF and SWF treatment compared to the SF and SLF treatment. Soil of the SF treatment showed a slightly negative CO_2_ budget of –35 kg CO_2_-C ha^-1^ compared to the S treatment. Cumulative CO_2_ emissions of the SLF treatment amounted to 202 kg CO_2_-C ha^-1^, in the SSF treatment to 1,613 kg CO_2_-C ha^-1^, and in the SWF treatment to 1,822 kg CO_2_-C ha^-1^ relative to the S treatment.

#### NO_3_^-^ Concentrations

Nitrification was the main source of NO_3_^-^ after application of ^15^N-fertilizer as indicated by a transfer of ^15^N signal from NH_4_^+^ to NO_3_^-^ (**Figures 1C,D**). Agricultural soil obtained from the experimental site had already initially a large NO_3_^-^-N background of 156 kg N ha^-1^ at 7 DBF (S treatment, **Figure 2F**). Application of wheat straw instantaneously and significantly reduced the soil NO_3_^-^ content by 69 kg N ha^-1^ at 7 DBF compared to the S treatment. Even after addition of extra mineral N of 150 kg N ha^-1^, NO_3_^-^ content in soil with wheat straw remained at a level of the S treatment without additional HCA or mineral N fertilization. Also spruce sawdust application tended to lower the NO_3_^-^ content in soil between 21 and 113 DAF, but to a lower extent compared to wheat straw. Lignin application did not reduce the NO_3_^-^ content and ranged around the same level as the SF treatment. During incubation, NO_3_^-^ content tended to increase in all treatments at the following rates: 0.7 (S), 1.3 (SF), 1.6 (SLF), 0.8 (SSF), 1.4 kg N ha^-1^ d^-1^ (SWF).

#### N_2_O Emissions

Like CO_2_ emissions, also N_2_O emissions were calculated for a 24-h time interval before soil sampling (**Figure 2G** and Supplementary Figure S2). Large N_2_O emissions occurred in all treatments after addition of NH_4_^+^-N as indicated at 7 DAF (**Figure 2D**). N_2_O emission tended to be lower in the SWF treatment at this time point compared to the SF, SLF and SSF treatments, which is in line with the rapid initial microbial N immobilization, especially in the SWF treatment. N_2_O emission patterns seemed to be influenced by the type of HCA, with a slight tendency to higher N_2_O emissions in the SSF and SWF treatment from 21 DAF onward. Nonetheless, N_2_O emissions dropped steeply at 21 DAF in most N-fertilized treatments, but with a delay in the SWF treatment. Cumulative N lost via N_2_O emissions from N-fertilized soil (SF) was 1.5 kg N ha^-1^ higher compared to the S treatment. The SWF and SLF treatment had both a cumulative N_2_O loss of 3.0 kg N ha^-1^, while the SSF treatment lost 4.0 kg N ha^-1^ in the form of N_2_O compared to the S treatment. In relation to the S treatment, the overall GWP (CO_2_+N_2_O) of soils with N fertilization (SF) or with additional HCA increased in the following order: 140 (SF) < 545 (SLF) < < 2,060 (SSF) < 2,162 (SWF) kg CO_2_-C equivalents ha^-1^.

## Discussion

### HCA-Derived N Immobilization

In our experiment, wheat straw, applied at a rate of 4.5 t C ha^-1^, increased N uptake by the microbial biomass up to 42 kg N ha^-1^ during a short period of time, before releasing two thirds of this amount again within the following 4 months of incubation at room temperature (**Figure 2D**). Such rapid N immobilization can also occur under field conditions after incorporation of organic C-rich crop residues (Congreves et al., 2013). Hence, HCA might be suitable to retain mineral N quickly in times of maximum mineral N availability, with a potential of dosed N release to germinating crops applied together with HCA. Some plants, such as cereal rye (*Secale cereale* L.), root down to a soil depth of 15 cm within 4 weeks after sowing, effectively reducing the mineral N loss (Brinsfield and Staver, 1991; Staver and Brinsfield, 1998) after its release from the microbial biomass. In contrast, winter wheat N uptake (30 kg N ha^-1^) is rather low during the winter season (Henke et al., 2008), but might be large enough to buffer the release of approximately 27 kg N ha^-1^ from the wheat straw-derived microbial biomass during 4 months and under conditions optimal for microbial activity.

Our data also indicates that different types of HCA, such as wheat straw and spruce sawdust, have the potential to retain 15 and 9 kg N ha^-1^ for more than 4 months, which, together with the N uptake of winter crops, might compensate N surpluses typically found in post-harvest fields (Döhler, 2009). In the medium term, N immobilized by microbial biomass may reduce N fertilizer demand in the following growing season, increasing the NUE of crop production. However, this requires that the N retained by the HCA is also released at the appropriate time and rate in order to transfer N to the next crop, which might be not easy to achieve under realistic field conditions as indicated by reduced yield and N uptake in the subsequent crop season and consequently lower profit margins (Thomsen and Christensen, 1998; Chaves et al., 2007, Congreves et al., 2013). Similar to the recommendations for appropriate mineral N application, choosing the right composition, amount, placement, timing (Bindraban et al., 2015), and the right particle size (Angers and Recous, 1997) may improve the applicability of HCA to retain N in soil until the next growth season. Hence, the N immobilization-release dynamics of this study might differ from field conditions in view of the microbially favorable conditions applied in this study (60% WHC, room temperature), but might be at least partly reached in the field by using a comparably small HCA particle size (Angers and Recous, 1997) like in our study, or by decreasing the depth of HCA incorporation into the soil (Christensen, 1986) in order to enhance microbial activities.

A fast N release from the wheat straw-derived microbial biomass might be related to the zymogenous microbial biomass as pioneers of the microbial wheat straw decomposer succession (Bastian et al., 2009) with rather short half-life of a few days (Cochran et al., 1988), and a rapid decay after exhaustion of easily accessible HCA components. This decay appears to have initiated a second microbial N immobilization maximum at 49 DAF (**Figure 2D**). In contrast, no such N immobilization-release dynamics were observed after application of spruce sawdust, which showed a more stable microbial N immobilization at 7 DAF and afterward (**Figure 2D**). Hence, microbial N release in soil with wheat straw could be combined with the spruce sawdust-derived microbial N immobilization. A combination of HCA with similar organic composition such as wheat straw and spruce sawdust might help to mitigate the fast microbial N re-mineralization after wheat straw application. Furthermore, adding HCA with more recalcitrant organic composition such as spruce sawdust and lignin potentially improves global agricultural C sequestration in soil.

In our experiment, microbial N immobilization-release dynamics (**Figure 2D**) were also paralleled by physical and / or chemical sequestration of about 24 kg N ha^-1^ in the remaining fraction of the wheat straw and spruce (**Figure 2B**). Also lignin was reported as matrix of chemical N fixation (Wei et al., 2017), particularly if reactive N forms like nitrite are present as demonstrated also in our study (**Figure 1F**). However, no significant N sequestration was found in soil with pure lignin application (**Figure 2B**). We assume that chemical N fixation by lignin is coupled to HCA-derived microbial dynamics. Miltner et al. (2012) provide strong evidence that disintegrating microbial populations are a relevant source of non-living soil organic matter. In accordance, we found indicators that a fast initial microbial growth based on easily available carbon derived from wheat straw was followed by such a rapid dieback of at least part of the microbial population. N derived from dead microorganisms, which does not directly serve as substrate for other microorganisms in the decomposer succession (Bastian et al., 2009), might be further transformed and fixed to recalcitrant organic molecules (Miyajima, 2015; Wei et al., 2017). Hence, N retained physically and / or chemically in the HCA matrix might be less available in the medium term, but incorporation of straw can improve crop yield and plant N uptake in the long term (Malhi et al., 2011). Overall, wheat straw (39 kg N ha^-1^) and spruce sawdust (33 kg N ha^-1^) induced microbial, physical and / or chemical N immobilization at field-relevant timescale and quantity, with clear potential to improve agricultural NUE and N retention capacity.

### Impact of HCA on Greenhouse Gas Release and Nitrate

Decomposition of HCA applied in our experiment, as prerequisite for microbial N immobilization, increased CO_2_ emission from soil over the runtime of the experiment (**Figure 2E**). Inversely to the HCA-derived CO_2_ emission, temporarily more C remained sequestered in soil with increasing recalcitrance of HCA to microbial decomposition. This makes spruce sawdust and lignin attractive to implement the recommendation of the French Ministry of Agriculture (2015), which aims to increase soil carbon stocks yearly by 4‰, helping to mitigate the adverse climatic influences of anthropogenic CO_2_ emissions. Mixed applications of HCA types such as wheat straw and spruce sawdust may be of benefit in order improve the temporal C sequestration and in parallel also the NUE. In 2010, global agricultural application on average reached a NUE of 0.42, but an increase by more than 60% (NUE of 0.68) is needed to secure the same food status for 9.1 billion people as assumed for the year 2050 (Zhang et al., 2015). Managing NO_3_^-^ seems particularly promising to improve NUE in crop production and N retention capacity, particularly in crop rotations with large potential N leaching losses of 44–73 kg N ha^-1^, such as winter crops following oilseed rape (Sieling and Kage, 2006). Our experiment showed a promising potential of wheat straw to instantaneously reduce the NO_3_^-^ concentration by 69 kg N ha^-1^ in soil with high mineral N background (**Figure 2F**), providing sufficient availability of C and N as a requirement of microbial growth and N immobilization (Fontaine et al., 2004). Application of HCA also showed a potential to mitigate NO_3_^-^ losses in cropping systems (Congreves et al., 2013), however, previously retained N appears to be lost at a later stage, e.g., in the following post-harvest season, from sandy loam soil with annual average precipitation and temperature of 862 mm and 7.7°C, respectively (Christensen, 1996; Thomsen and Christensen, 1998).

Despite continuous NO_3_^-^ release of re-mineralized microbial N during the entire incubation, wheat straw, and less pronounced and delayed also spruce sawdust, tended to reduce soil NO_3_^-^ content at a field-relevant time scale. The observed NO_3_^-^ mitigation might be a result of outcompeting nitrifiers and denitrifiers by zymogenous N immobilizers (Burger and Jackson, 2003), as indicated by our data (**Figure 3**). Hence, HCA such as wheat straw and spruce sawdust with high C content have the potential to mitigate NO_3_^-^ losses if applied to soil in the right amount and under conditions suitable for large microbial competition for mineral N.

Another major source of critical N losses in agriculture with negative impact on NUE and climate change is the emission of N_2_O (Smith, 2017). In our experiment, spruce sawdust and wheat straw, as promising substrates for N immobilization, did not decrease N_2_O emission (**Figure 2G**). Most of the N_2_O emission was associated with the mineral NH_4_^+^ excess directly after fertilization. Ammonia oxidation, as part of nitrification, likely was the main source of N_2_O emissions in our well-aerated soil (Mathieu et al., 2006). Transfer of the ^15^N signal from the fraction of NH_4_^+^ to NO_3_^-^ (**Figures 1C,D**) also underlines the importance of nitrification for the observed N_2_O emissions after fertilization with NH_4_^+^, which as an autotrophic process is fairly independent from the type of organic carbon amendment. Also nitrification after mineralization of N-rich crops such as lettuce can cause large N_2_O emissions of 1.1 kg N ha^-1^ within a short period of time (Baggs et al., 2000). NO_3_^-^ as potential substrate for denitrification was not limited during the entire incubation period and obviously had no relevant influence on N_2_O emissions from our well-aerated soil. Nonetheless, remaining NO_3_^-^ might be problematic in the winter season under field conditions (Kaiser and Ruser, 2000), particularly in almost water-saturated soils after fertilization, after incorporation of crop residues, but also after weather-induced pulses of N mineralization (Liang et al., 2016).

Microbial N turnover occurs in soils particularly during times of microbial biomass decay, providing mineralized N to nearby nitrifiers and denitrifiers. Thus, only HCA with high C availability, such as wheat straw, temporarily tend to reduce nitrification-derived N_2_O emissions (**Figure 2G**) by increasing the microbial competition of N_2_O-producers with fast growing microorganisms with large N immobilization rates (Burger and Jackson, 2003). Nonetheless, our data shows that this only leads to an increase of N_2_O emission at a later stage, when part of the HCA-derived microbial biomass becomes mineralized again.

A complete denitrification of N_2_O to N_2_ might be another option to reduce problematic N_2_O emissions (Zhang et al., 2013). In the mid-term, soil with spruce sawdust induced a larger abundance of denitrifiers harboring the *nosZ* gene, which codes the transformation of N_2_O to N_2_, however, not early enough to mitigate the initial N_2_O peak. Hence, soil amended with spruce sawdust emitted more N in form of N_2_O compared to all other soil treatments in our study, resulting in an overall GWP of CO_2_ and N_2_O emissions from soils with spruce sawdust comparable to wheat straw, which in relation released more CO_2_, but less N_2_O. We assume that HCA, such as wheat straw and sawdust, do not have a great potential to mitigate N_2_O emission at a field-relevant scale, which is in line with previous findings (Frimpong and Baggs, 2010).

## Conclusion

We hypothesized that HCA such as wheat straw, spruce sawdust, and lignin have the potential to manage temporal N excess by inducing microbial growth at a field-relevant scale. We conclude that HCA based on wheat straw or similar material can be particularly effective in buffering an excess of N at a field-relevant scale in a short period of time (days), as often reported for post-harvest sugar beet and oilseed rape fields. N immobilization benefits might be optimized by combining HCA with properties similar to wheat straw and spruce sawdust. In our experiment, wheat straw and spruce sawdust were capable of retaining N long enough within the microbial and HCA residue pool to serve as additional N supply for the following crop growth next spring. A prerequisite of N immobilization is microbial activation by HCA, which cannot be induced by applying very recalcitrant HCA types such as lignin. Furthermore, we hypothesize that HCA mitigates N losses in form of NO_3_^-^. Application of HCA such as wheat straw and spruce sawdust to mineral N-rich soil temporarily increases CO_2_ and N_2_O emissions, which were, however, counterbalanced by advantages such as N immobilization and mitigation of the NO_3_^-^ loss potential. We conclude that HCA applications can be part of a whole set of novel agricultural cultivation strategies to improve NUE of agricultural production, without losing agricultural productivity and sustainability. However, further research is needed to optimize HCA amount, composition, and particle size for particular crop rotations and N fertilization regimes to ensure appropriate immobilization and re-release of N at the right time and at the required rate.

## Author Contributions

RR and NB wrote the manuscript. RR, JW, and NB designed the study. RR, MI, and JW performed the sampling. MI installed and maintained the experiment. RR provided information and performed the microbial biomass measurement. RR, JW, and NB calculated and reviewed the greenhouse gas data. HW performed the N liquid-liquid extractions and the δ^15^N measurements. CS performed the quantitative real time PCR on the abundance of nitrifiers and denitrifiers. CS, PS, and MS reviewed the real time PCR results and the manuscript. RR, JW, MI, CS, HW, PS, MS, and NB revised the manuscript.

## Conflict of Interest Statement

The authors declare that the research was conducted in the absence of any commercial or financial relationships that could be construed as a potential conflict of interest.
