# Supplementary material for: Potential of Wheat Straw, Spruce Sawdust, and Lignin as High Organic Carbon Soil Amendments to Improve Agricultural Nitrogen Retention Capacity: An Incubation Study
Source: Front Plant Sci. 2018 Jun 28;9:900. doi: 10.3389/fpls.2018.00900 (PMC6031754; doi:10.3389/fpls.2018.00900)
Supplement: Supplementary file 1 [file Data_Sheet_1.PDF]

Supplementary data

Potential of wheat straw, spruce sawdust, and lignin as high organic carbon soil amendments to improve agricultural nitrogen retention capacity: an incubation study

Rüdiger Reichel[1], Jing Wei[1], Muhammad Saiful Islam[2], Christoph Schmid[3], Holger Wissel[1], Peter Schröder[3], Michael Schloter[3], Nicolas Brüggemann[1]

[1] Forschungszentrum Jülich GmbH, Institute of Bio- and Geosciences, Agrosphere (IBG-3), 52425 Jülich, Germany
[2] University of Bremen, General and Theoretical Ecology, Institute of Ecology, 28359 Bremen, Germany
[3] Helmholtz Zentrum München, Deutsches Forschungszentrum für Gesundheit und Umwelt GmbH, Research Unit Comparative Microbiome Analysis (COMI), 85764 München, Germany

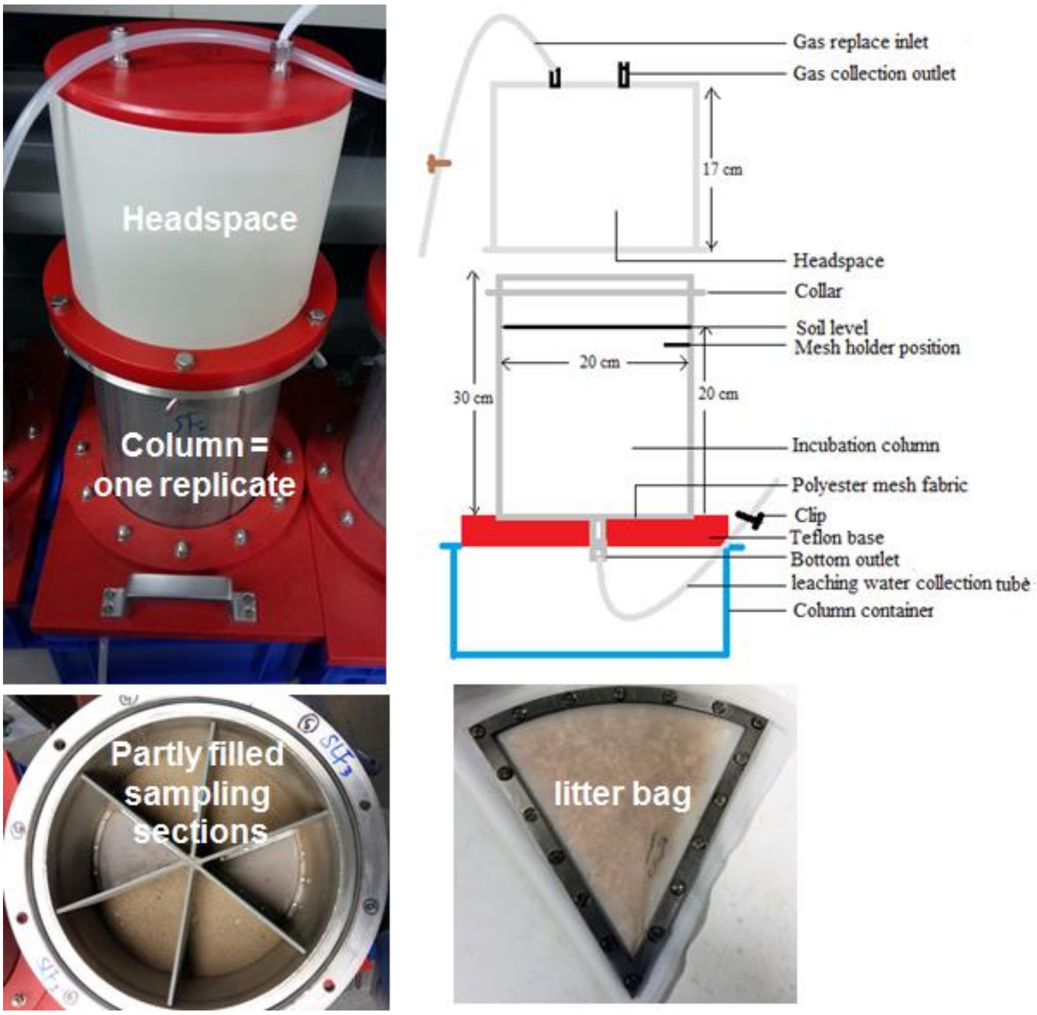

Figure S1. Stainless steel incubation column with headspace unit (top left); sketch of the incubation system (top right); segmentation for soil sampling at six incubation times (lower left); nylon mesh litter bags (pore size 0.2 µm; lower right).

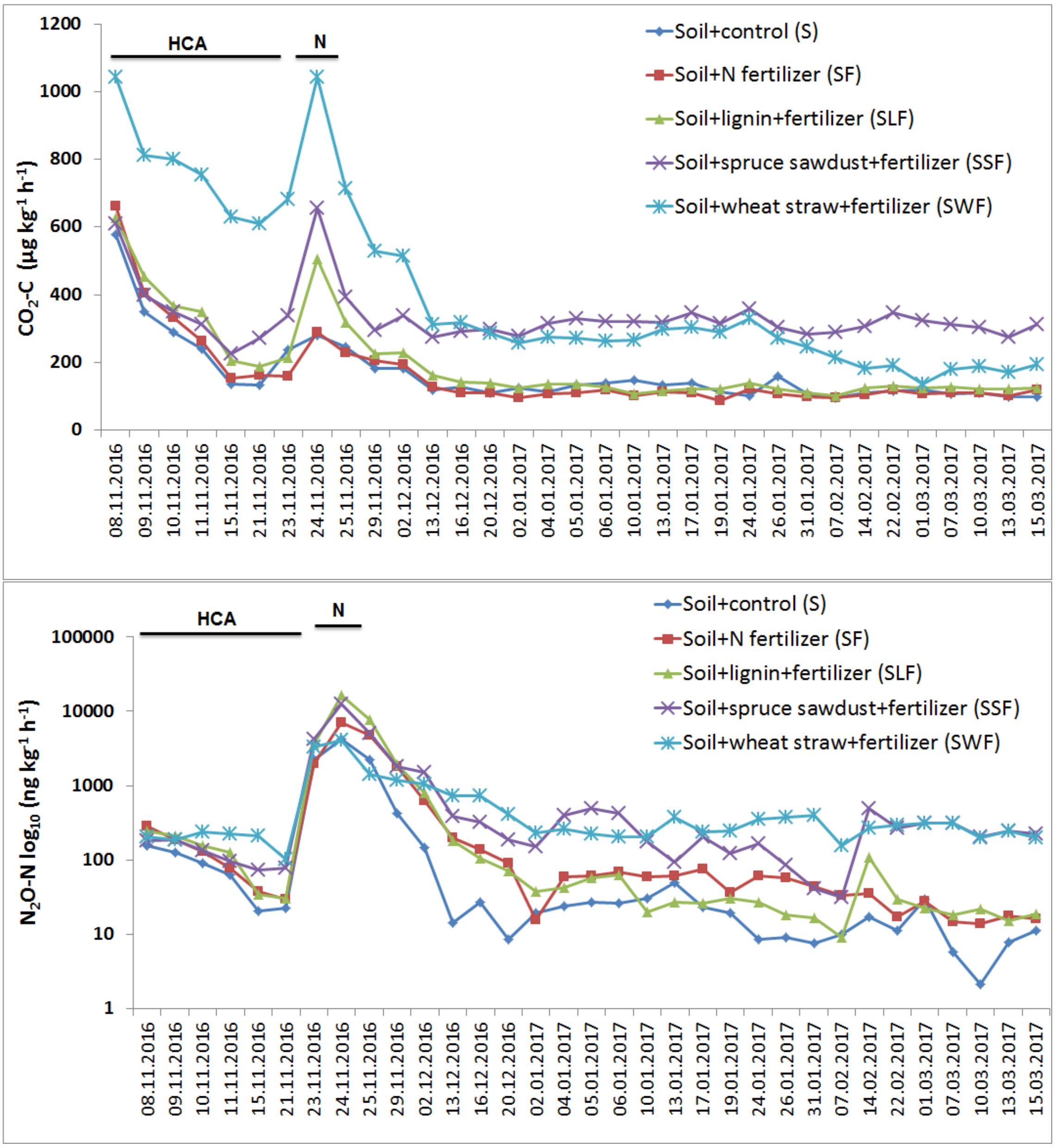

Figure S2. Mean CO_2 (µg C kg^{-1} h^{-1}) and N_2O emission (log scale, ng N kg^{-1} h^{-1}) in soil of the soil control treatment (S) without any N fertilizer (N) or high organic carbon soil amendments (HCA), the fertilizer control treatment (SF) with mineral N fertilizer, and the HCA treatments with mineral N fertilizer plus lignin (SLF), spruce sawdust (SSF), or wheat straw (SWF). Incubation time d = 0 divides the experiment into a period before (DBF) and after mineral N fertilization (DAF). Soil sampling was conducted only at 7 DBF (16 Nov), 7 DAF (30 Nov), 21 (14 Dec), 49 (11 Jan), 77 (8 Feb), and 113 DAF (16 Mar).
